# Complete chloroplast genome sequence of *Lirianthe coco* (Loureiro) N. H. Xia & C. Y. Wu (Magnoliaceae), a popular ornamental species

**DOI:** 10.1080/23802359.2020.1775143

**Published:** 2020-06-11

**Authors:** Yongkang Sima, Tao Wu, Yupin Fu, Jiabo Hao, Shaoyu Chen

**Affiliations:** Yunnan Academy of Forestry & Grassland, Kunming, Yunnan, People’s Republic of China

**Keywords:** Magnoliaceae, *Lirianthe coco* (Loureiro) N. H. Xia & C. Y. Wu, complete chloroplast genome, phylogenetic analysis

## Abstract

*Lirianthe coco* (Loureiro) N. H. Xia & C. Y. Wu is a popular ornamental species of Magnoliaceae. In the present study, the complete chloroplast genome (cpDNA) of *L. coco* was sequenced, assembled, and analyzed. The results indicated that the size of chloroplast genome of *L. coco* is 159,828 bp, which exhibits a typical quadripartite structure including a large single-copy (LSC) region of 87,958 bp and a small single-copy (SSC) region of 18,768 bp separated by a pair of identical inverted repeat regions (IRs) of 26,551 bp each. The genome contained 131 genes (113 unique), including 86 protein-coding genes (80 unique), 37 tRNA genes (29 unique), and 8 rRNA genes (4 unique). Phylogenetic analysis showed that *L. coco* is affinal to *L. odoratissima* and forms a nomophyletic group with the latter and *L. delavayi*.

*Lirianthe coco* (Loureiro) N. H. Xia & C. Y. Wu is a species of the genus *Lirianthe* Spach within the family Magnoliaceae, native to Fujian, Guangdong, Guangxi, Taiwan, Yunnan, Zhejiang of China and Vietnam. It is grown as an ornamental shrub or tree in popular and widely used in gardening (Lin et al. 2007). *Lirianthe coco* (Loureiro) N. H. Xia & C. Y. Wu is cultivated as a plant for perfume and used medicinally (Rui et al. 1991; Wang et al. 2014; Sima et al. 2018). However, there has been no genomic study on *Lirianthe coco* (Loureiro) N. H. Xia & C. Y. Wu.

In the study, we reported the complete sequence of chloroplast genome of *Lirianthe coco* (Loureiro) N. H. Xia & C. Y. Wu. The GenBank accession number is MT225530. The leaf sample of a shrub of *Lirianthe coco* (Loureiro) N. H. Xia & C. Y. Wu was collected from Kunming Arboretum, Yunnan Academy of Forestry & Grassland Science, Yunnan Province of China (25°9′4″N, 102°44′45″E). The sheets of vouchered specimen (Y. K. Sima & Shaoyu Chen, 99279) are deposited at the herbaria of YAF and YCP. Total DNA was extracted from the collected fresh leaves using DNA Plantzol Reagent (Invitrogen, Carlsbad, CA, USA).

Genome sequencing was performed using Illumina HiSeq Sequencing System (Illumina, San Diego, CA) to construct the shotgun library. About 1.5 G pair-end (150 bp) raw sequence data were obtained and the low-quality sequences were filtered through CLC Genomics Workbench v8.0 (CLC Bio, Aarhus, Denmark) to get high-quality clean reads. *Pachylarnax sinica* (Y. W. Law) N. H. Xia & C. Y. Wu (JX280400) served as the reference, NOVO Plasty software (Dierckxsens et al. 2017) was used to align and assemble cp genome. The genome was automatically annotated using CpGAVAS (Liu et al. 2012) and then adjusted and confirmed with Geneious 9.1 (Kearse et al. 2012). The annotated genomic sequence was submitted to GenBank. In order to better determine the phylogenetic position of *Lirianthe coco* (Loureiro) N. H. Xia & C. Y. Wu, the complete chloroplast genome sequences of other 26 species of the subfamily Magnolioideae from NCBI were aligned using MAFFT v. 7 (Sima and Lu 2012; Katoh and Standley 2013; Sima et al. 2014). Based on the system of Magnoliaceae by Sima and Lu (2012), two species of the subfamily Liriodendroideae, *Liriodendron chinense* (Hemsley) Sargent (KU170538) and *Liriodendron tulipifera* Linnaeus (DQB99947) were served as the outgroup. The maximum likelihood (ML) tree was reconstructed with RAxML (implemented in Geneious ver.10.1 http://www.geneious.com, Kearse et al. 2012) and bootstrap probability values were calculated from 1000 replicates.

The size of the complete assembled chloroplast genome of *Lirianthe coco* (Loureiro) N. H. Xia & C. Y. Wu is 159,828 bp, which exhibits a typical characteristics in its general structure with a large single-copy (LSC) region of 87,958 bp and a small single-copy (SSC) region of 18,768 bp separated by a pair of identical inverted repeat regions (IRs) of 26,551 bp each. A total of 113 genes were successfully annotated containing 79 protein-coding genes, 30 tRNA genes, and 4 rRNA genes. The result of phylogenetic analysis showed that *Lirianthe coco* (Loureiro) N. H. Xia & C. Y. Wu is affinal to *Lirianthe odoratissima* (Y. W. Law & R. Z. Zhou) N. H. Xia & C. Y. Wu (MH795108) and formed a nomophyletic group with the latter and *Lirianthe delavayi* (Franchet) N. H. Xia & C. Y. Wu (MN780910, MN783014) (Figure 1), and this *Lirianthe* clade is sister to the *Talauma* clade with high support. All genera mentioned in this analysis are nomophyletic under the taxonomical system of Magnoliaceae by Sima and Lu (2012). The study would provide effective new molecular data for evolutionary and phylogenetic analysis of Magnoliaceae.

**Figure 1.**
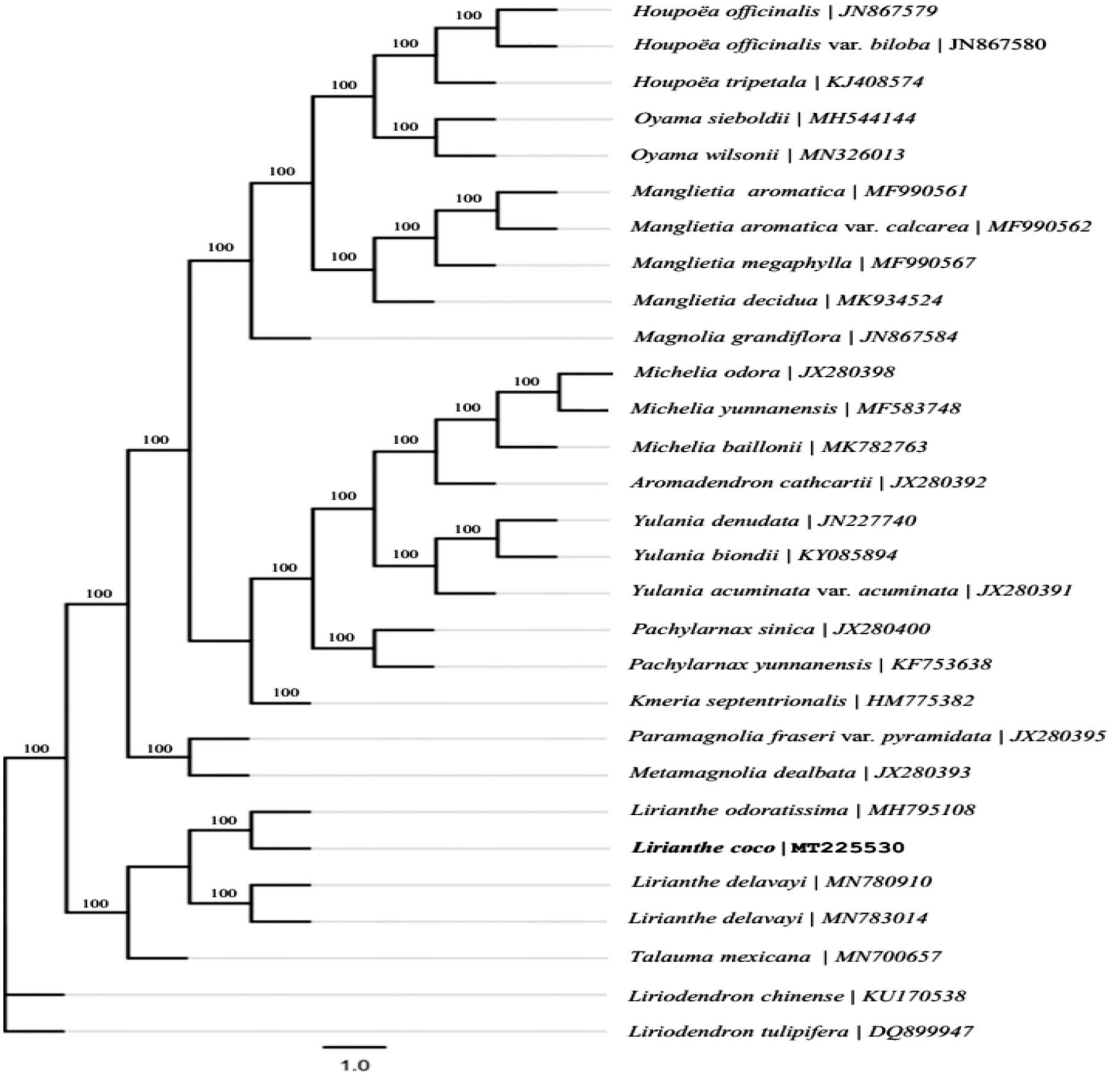
The maximum-likelihood tree based on the chloroplast genomes of 26 species of Magnolioideae and 2 species of Liriodendroideae in the family Magnoliaceae. Bootstrap values (1000 replicates) are shown at the nodes.

## Data Availability

Scientific name of the organism in the paper: *Lirianthe coco* (Loureiro) N. H. Xia & C. Y. Wu. Geographic location of the specimen: Kunming Arboretum, Yunnan Academy of Forestry & Grassland Science, Yunnan Province of China (25°9′4″N, 102°44′45″E). The specimen is stored at the herbaria of Yunnan Academy of Forestry & Grassland Science, Yunnan Province of China The cpDNA sequence of *Lirianthe coco* (Loureiro) N. H. Xia & C. Y. Wu that support this study are openly available in The National Center for Biotechnology Information (NCBI) > DNA & RNA > Nucleotide Database at https://www.ncbi.nlm.nih.gov. GenBank accession number is MT225530.
